# A Medical Secretary of Public Health

**Published:** 1892-04

**Authors:** 


### SECRETARY OF PUBLIC HEALTH.

Senator Sherman has introduced in Congress a bill to create a Department of Public Health, with a Medical Secretary as executive officer, in pursuance of a request from the American Medical Association,—a bill which we think "fills the bill." Whether the bill will be passed, whether it will be amended out of all recognition, or whether any bill of the kind will go through remains to be seen. The medical profession are clamoring for it in the interest of public health, and Congress will hardly ignore so emphatic expression of sentiment, practically unanimous, of the profession of America. The need of such a bill is manifest; self-evident.

Assuming, therefore, that some such measure will be adopted; that a Secretary will have to be appointed, the JOURNAL ventures to predict now the appointment of Dr. John B. Hamilton. It lays odds that he is the coming Secretary. His work in organizing and administering the great Marine Hospital Service several years ago stands as a monument to his administrative and executive ability. His knowledge of general medicine is extensive, as evidenced by his great address on the history of medicine; while of special medicine and surgery he is a master. He is young and physically strong; and, we want no "has been" in the pilot-house. We are aware that he has enemies in the profession. What brilliant and progressive (or aggressive) man has not? That there are leading men in the American Medical Association who would rather see the country gone to the bow-wows than to see Dr. Hamilton exalted to this position; but, all the same, he will get there. In the judgment of the JOURNAL he is eminently qualified for the place, and has claims which the Republican administration will recognize, notwithstanding his inability to oust Dr. Wyman as Surgeon General.

We do not say he is our choice for the position; far from it. Nor that he is the only man able to fill it. There is one man, in particular, who, in our judgment, would make as good—we will not say better, Secretary than he, and who would bring to the position as much dignity, ability and knowledge as any man in America, and who would add to these qualities a high born courtesy of manner, both in official and social intercourse, possessed by few men. We refer to our own State Health Officer of Texas, R. M. Swearingen. He would be an ornament to the position and would fill the vast and intricate requirements of the position with distinguished ability. He has given evidence of the highest order of talent in sanitary administration; but he must be regarded as *hors du concours* so long as the Republicans have charge of the government. But, nevertheless, we predict that the appointment will fall to the able and aggressive young medical politician John B. Hamilton,—notwithstanding he is accused of killing the National Board of Health of 1879–82.

